# The Durban University of Technology Faculty of Health Sciences Decentralized Clinical Training Project: Protocol for an Implementation Study in KwaZulu-Natal, South Africa

**DOI:** 10.2196/52243

**Published:** 2024-06-03

**Authors:** Celenkosini Thembelenkosini Nxumalo, Pavitra Pillay, Gugu Gladness Mchunu

**Affiliations:** 1 Academic Development Unit Faculty of Health Sciences Durban University of Technology Durban South Africa; 2 Office of the DVC Research and Innovation Research Development and Postgraduate Support University of the Western Cape Cape Town South Africa; 3 Faculty of Health Sciences Durban University of Technology Durban South Africa

**Keywords:** decentralized clinical training programs, curriculum, clinical education, health science education, university of technology, pedagogy, transformative education, teaching, higher education, South Africa

## Abstract

**Background:**

The Durban University of Technology (DUT) Faculty of Health Sciences (FHS) in KwaZulu-Natal, South Africa, is embarking on a project to implement a Decentralized Clinical Training Program (DCTP). The DUT FHS DCTP project is being conducted in response to the growing demands of students requiring clinical service placements as part of work-integrated learning. The project is also geared toward responding to existing gaps in current practices related to the implementation of a DCTP, which has mainly been through traditional universities providing training to medical, optometry, occupational therapy, and physiotherapy students. In South Africa, a DCTP is yet to be implemented within the context of a university of technology; it is yet to be implemented within health science faculties that offer undergraduate health science programs in mainstream biomedicine and alternative and complementary disciplines.

**Objective:**

We aim to design, pilot, and establish an effective DCTP at the DUT FHS in KwaZulu-Natal, South Africa.

**Methods:**

Participatory action research comprising various designs—namely, appreciative inquiry, qualitative case study design, phenomenography, and descriptive qualitative study design—will be used to conduct the study. Data will be collected using individual interviews, focus group discussions, nominal group technique, consensus methodology, and narrative inquiry. Study participants will include various internal and external stakeholders of the DUT, namely, academic staff; students; key informants from universities currently using successfully established DCTPs; academic support staff; staff working in human resources, finance, procurement, and accounting; and experts in other disciplines such as engineering and information systems. Overall, 4 undergraduate health science programs—namely, Radiography, Medical Orthotics and Prosthetics, Clinical Technology, and Emergency Medical Care and Rescue—will be part of the project’s pilot phase. Findings from the project’s pilot phase will be used to inform scale-up in the other undergraduate programs in the DUT FHS. The project is being implemented as part of the university’s strategic objective of devising innovative curricula and pedagogical practices to improve the mastery, skill set, and competence of health science graduates.

**Results:**

The study has currently commenced with the situational analysis, consisting of engagement with external stakeholders implementing DCTPs. The data to be generated from the completion of the situational analysis are anticipated to be published in 2024.

**Conclusions:**

This project is envisioned to facilitate collaboration among the universities of technology, traditional universities, Ministry of Health, and private sector for clinical placement of undergraduate health science students in health establishments that are away from the university, thereby exposing them to real-life experiences related to health care. This will facilitate authentic learning experiences that will contribute to improved competencies of graduates in relation to the health needs of society and the multiple realities of the South African health system.

**International Registered Report Identifier (IRRID):**

PRR1-10.2196/52243

## Introduction

### Background

Decentralized Clinical Training Programs (DCTPs) refer to learning platforms adopted in health science education, which facilitate theoretical and clinical learning in remote decentralized health facilities that are often some distance away from traditional academic teaching health establishments [1]. Traditional DCTPs have predominantly been implemented with medical students through the use of community-based primary health care facilities, community health centers, and district hospitals [2-4]. Facilitating successful implementation of DCTP for health science education requires a geographical shift in terms of clinical placement facilities used for work-integrated learning of students. Moreover, a curriculum and pedagogical shift from the traditional teaching approach is necessary to maximize the benefits of this strategy [5]. Initial implementation of DCTP in low- to middle-income countries such as South Africa predominantly involves a geographical shift; however, developments in recent years have resulted in universities adopting both a geographical and pedagogical shift due to the many potential benefits of such a change. By facilitating the pedagogical shift, higher education institutions implementing DCTP have realigned health science curricula to place a strong emphasis on the primary health care approach [6,7]. Thus, this serves to align health science education practices with the current evolving nexus of higher education and the changing landscape of health service delivery necessitated by developments in global and regional health policies [8,9].

DCTPs are considered to be transformative approaches to health science education adopted by higher education institutions, in response to the growing demand for clinical service placement owing to the increasing number of students being registered for medical and health disciplines [10,11]. Studies further suggest that the benefits of DCTPs include fostering a sense of social accountability through clinical placement of students in community-based settings and rural health facilities [12]. This process also exposes undergraduate students to the multiple realities of the health systems and the varying social determinants of health. Training students through DCTP produces successful graduates who are more fit for purpose in terms of community needs and the regional landscape of health service delivery [13].

Studies conducted on existing implementation models of DCTPs, particularly in South Africa, have suggested that this approach to health science education has predominantly been implemented in traditional universities. In recent years, students taught through DCTPs have been expanded beyond medicine to include undergraduate students in the multidisciplinary biomedical system of health care, which encompasses nursing, occupational therapy, physiotherapy, and optometry, to name a few. Anecdotal evidence regarding students’ experiences of and perspectives about DCTPs indicates overall positive learning experiences reported by students who are taught using this approach [14,15]. Moreover, students have perceived education through DCTPs as strengthening their responsiveness, preparedness, and relevance as graduates. Similar studies about educators’ experiences of DCTPs have also reported positive perspectives regarding the value of teaching and learning using this platform [16]. Additional studies regarding the perspectives about DCTPs have also been elicited from other stakeholders such as service providers and public health service managers. A study to explore public health sector optometrists’ perspectives regarding a DCTP in KwaZulu-Natal, South Africa, revealed that while the DCTP was generally supported by the optometrists, challenges were reported in relation to the resources required for comprehensive optometric assessments. Despite the challenges cited, the study highlighted the DCTP as an important strategy for improving eye care service capacity in KwaZulu-Natal, South Africa [17].

In South Africa, health services are rendered through a re-engineered primary health care approach using the district health system as a vehicle to drive primary health care services [18,19]. Moreover, the newly adopted National Health Insurance (NHI) bill serves to further propel the agenda of universal health coverage, consistent with the sustainable development goals. The current NHI bill makes provision for public and private health sector collaborations to ensure optimum service delivery for all, irrespective of socioeconomic status. Thus, this change in health policy reform requires health care workers who are adequately prepared in terms of the various aspects of the health system. Therefore, the nature of teaching and learning using DCTPs is instrumental in facilitating the realization of this prepared state using health science education [20]. Moreover, the globalization of health care calls for the adoption of such innovative approaches in curriculum and pedagogy so that graduates are equipped to deal with the multitude of health demands arising from policy and health practice changes.

The benefits of DCTPs as an adaptive methodology for teaching and learning within health sciences cannot be overemphasized. Thus, there is increasing advocacy for the adoption of DCTPs in health science education; moreover, there is growing evidence regarding the models to facilitate the implementation of DCTPs, particularly within the South African higher education institutions offering medical and related health qualifications that form a part of mainstream biomedicine. Despite growing evidence in support for DCTPs from various categories of stakeholders—namely, students, health educationists, and global policy development experts—gaps persist in the practice of implementing DCTPs in higher education institutions in South Africa. Moreover, the full complement of health science disciplines has yet to implement this teaching approach at higher education institutions.

In South Africa, DCTP implementation is currently limited to certain traditional universities that have implemented DCTPs in specified health science disciplines such as medicine, occupational therapy, optometry, and physiotherapy [12,21]. Universities of technology (UoTs) are yet to implement DCTPs within health science disciplines offered within such institutions. Principally, UoTs are a new classification of technikons that previously offered diploma qualifications while harnessing technological advancement and partnerships with society and industry to solve community problems [22]. UoTs differ from traditional universities in that their primary focus is vocational and technical education while responding to a social responsibility of promoting access, redress, and equity. UoTs are driven by market forces and entrepreneurialism, whereas traditional universities’ purpose is motivated by ideation and liberalism [23]. In the context of implementing a DCTP within a UoT, which will be a first in South African higher education history, the key areas of differences will be the overarching objective of producing graduates who are responsive to the health needs of the communities and society in the South African region. This will be facilitated through the learning that will allow health science students to be exposed to diverse, real-life, health-related experiences and challenges in their natural context. Another key difference will be the exposure to diverse health conditions and scenarios during the clinical placement that is not limited to a specific type of health setting. In this regard, students will be placed in health facilities that encompass the broad range of spheres within the national health system. Students’ clinical exposure will encompass private and public health facilities across all levels, commencing from community-based health care to regional-level or tertiary-level health services. The envisaged partnership among the UoTs, traditional universities, private health sector, and public health sector will be facilitated through strategic high-level engagement with relevant authorities and will be operationalized through a documented memorandum of understanding among the institutions.

Considering the gap in practice and the reported benefits of DCTPs in terms of health science graduates’ competencies and preparedness for the work environment, the Durban University of Technology (DUT) seeks to design, pilot, and evaluate a model to facilitate the successful establishment of a DCTP within the dimensions of contextual factors influencing teaching and learning at a UoT. In their position paper about the implementation of a DCTP at a UoT, the institution seeks to use a pilot of selected health disciplines to inform the design, piloting, and establishment of a DCTP, which will eventually be rolled out to the Faculty of Health Sciences (FHS), based on the outcomes of the pilot phase of this project [24]. While the implementation of a DCTP within the UoT will be a first in South African higher education practices, many challenges are anticipated in relation to piloting and implementation. These challenges relate to the availability of resources such as financial capital, human resources, and physical infrastructure. Other anticipated challenges relate to student and staff perceptions and existing challenges that are associated with clinical practice in remote areas, particularly those that are underresourced. Therefore, the UoT plans to establish collaborations with existing universities that are implementing DCTPs and engage with various categories of stakeholders’ prior piloting and implementation and scale-up of the project. An iterative process of data generation facilitated by multistakeholder engagement will be performed throughout all phases of the project as part of monitoring and evaluation to ensure success of the project at all stages.

The DUT currently consists of 6 faculties, which include the FHS. The FHS offers undergraduate and postgraduate qualifications in health sciences that lead to the professional development of graduates as health professionals in different fields of health sciences. The faculty currently has the following academic departments: Biomedical and Clinical Technology, Community Health Studies, Chiropractic, Dental Sciences, Emergency Medical Care and Rescue, Medical Orthotics and Prosthetics, Nursing, Radiography, Homoeopathy, and Somatology. The faculty offers a range of courses within these departments, which lead to the development of health professionals for mainstream and alternative health care. For the purpose of design and testing, the undergraduate academic departments that will form a part of the pilot are Radiography, Emergency Medical Care and Rescue, Clinical Technology, and Medical Orthotics and Prosthetics. While the envisaged methodology is discussed further in this paper, the execution of the project remains relatively subject to change, given the diversity of the courses included in the pilot and the nature of teaching and learning within a UoT. Nonetheless, the project will be valuable, as the institution’s FHS will be the first UoT to pilot and implement a DCTP within the context of health science education, which encompasses biomedical and alternative health care disciplines. The findings of the proposed project have implications for research, education, and practice related to DCTPs, both from a broad higher education perspective and within contextual factors related to UoTs.

### Aim

We aim to design, pilot, evaluate, and establish an effective DCTP at a selected UoT in KwaZulu-Natal, South Africa.

### Objectives

This study has the following objectives:

1. To critically analyze the current practice of implementing a DCTP at selected higher education institutions in South Africa
2. To explore stakeholders’ perceptions regarding the range of factors to be considered to design, pilot, and establish a successful DCTP at a selected UoT in KwaZulu-Natal, South Africa
3. To design, pilot, and establish systems to aid the implementation of a DCTP at a selected UoT in KwaZulu-Natal, South Africa
4. To propose a relevant model for the successful establishment of a DCTP at a selected UoT in KwaZulu-Natal, South Africa

### Research Questions

Following is the research question in relation to objective 1:

1. What is the current practice of implementing DCTPs at selected higher education institutions in South Africa?

Following are the research questions in relation to objective 2:

1. What are the stakeholders’ perceptions regarding the range of factors to be considered in designing, piloting, evaluating, and establishing a successful DCTP at a selected UoT in KwaZulu-Natal, South Africa?
2. What are the stakeholders’ perceptions about the range of infrastructure, pedagogy, curriculum, and human resources factors that are related to the successful establishment of DCTP at a selected UoT in KwaZulu-Natal, South Africa?

Following are the research questions in relation to objective 3:

1. What approach or model can be designed or adopted to pilot, evaluate, and establish a successful DCTP at a selected UoT in KwaZulu-Natal, South Africa?
2. What are the range of IT systems required to be designed, piloted, and established to support the successful testing, evaluation, and establishment of a DCTP at a selected UoT in KwaZulu-Natal, South Africa?
3. What are the range of human resource and financial systems required to be designed, piloted, and established to support the successful testing, evaluation, and establishment of a DCTP at a selected UoT in KwaZulu-Natal, South Africa?
4. What are the range of infrastructure systems that need to be designed, piloted, and established to support the successful testing, evaluation, and establishment of DCTP at a selected UoT in KwaZulu-Natal, South Africa?
5. What are the curriculum and pedagogical systems required to be designed, piloted, and established to support the successful testing, evaluation, and establishment of a DCTP at a selected UoT in KwaZulu-Natal, South Africa?
6. What are the stakeholders’ experiences, understanding, and perceptions regarding the design, testing, and establishment of a DCTP at a selected UoT in KwaZulu-Natal, South Africa?

Following is the research question in relation to objective 4:

1. What intervention model may be proposed to establish a successful DCTP at a selected UoT in KwaZulu-Natal, South Africa?

### Context of the Study

The study will be conducted within the KwaZulu-Natal province of South Africa. The DUT will be one of the primary data collection sites because this is where the DCTP will be designed, piloted, and evaluated for successful establishment. This design process will be informed by a multistakeholder engagement process, which includes consultation with individuals who are internal and those who are external to the UoT. As the University of KwaZulu-Natal (UKZN) has already successfully established several decentralized clinical platforms in KwaZulu-Natal, the university and personnel affiliated to these platforms will be key informants in this project. Thus, the DUT plans to formalize a partnership with the UKZN for the purpose of executing this project.

### Theoretical and Conceptual Underpinnings

Theories of health science education practice and concepts related to DCTP implementation as identified by the literature will be refined and operationalized to guide this study. Moreover, existing policies related to curriculum and pedagogy, as governed by the UoT’s strategic objectives, guidelines, and related polices, will form a part of the conceptual map to guide the study. The conceptual framework and theories to be used are in the process of being refined and have been used to develop the research objectives and questions. These will subsequently guide the inquiry process of this study.

## Methods

### Overview

A multimethod design encompassing quantitative and qualitative research approaches, using different research designs underpinned by participatory action research, will be used to conduct the study. This design will allow for the use of multiple forms of quantitative and qualitative approaches to collect data from various sources through different forms of data collection (surveys, individual in-depth interviews, focus group discussions, consensus-building sessions, and implementation science processes). The multimethod design will be underpinned by participatory action research because the broad aim of the study relates to intervention design, testing, and evaluation. The study will be executed in various phases, namely, (1) situational analysis; (2) empirical phase (piloting and preliminary testing); (3) evaluation and refining; and (4) establishment and continuous monitoring, as underpinned by the abovementioned design. The phases of execution for the proposed project are discussed in the following sections.

### Phase 1: Situational Analysis

#### Objective

The purpose of the inquiry phase is to develop a conceptual framework and understanding to guide the process of developing, piloting, evaluating, and establishing an effective DCTP at a selected UoT in KwaZulu-Natal, South Africa. Then, the conceptual model will inform the development of a logic model with a set of activities to operationalize key elements in the conceptual framework that is focused upon the broader aim of piloting and implementing the DCTP. The inquiry phase will also comprise high-level engagement with key informants to determine the primary factors for consideration when piloting, implementing, and evaluating the DCTP at the selected UoT. Thus, the following methods and related approaches will be included in phase 1.

#### Step 1: Desktop Review

This step will encompass the review of existing literature and research about the models and reported experiences of implementing DCTPs in higher education institutions, with the review of best practices, international perspectives, and evidence from low- to middle-income countries. The review will be conducted using systematic scoping, narrative review, and integrative methods. The desktop review will also include a review of relevant policies and guidelines related to teaching and learning, higher education practices, and related phenomena.

#### Step 2: Stakeholder Engagement and Benchmarking

This step will be underpinned by participatory action research design, wherein various categories of key informants will be consulted to conceptualize the strategy of designing, piloting, and implementing DCTPs. In this step, engagement sessions will be facilitated through workshop sessions to obtain buy-in from executive-level stakeholders, with training and evaluation embedded within each session. A combination of focus group discussions and individual in-depth interviews will be used to collect data during the stakeholder engagement sessions. On the basis of the emerging findings from individual interviews and focus group discussions, consensus methodology will be used to triangulate the data that will respond to key questions of the research project and inform the emerging phases of the project. For this purpose, a combination of qualitative research designs will be used, namely, descriptive qualitative design, case study design, and appreciative inquiry, as part of the participatory action research design. The stakeholder engagement workshop sessions will also be designed using appreciative inquiry to stimulate discussion around the key factors to be considered during the design, pilot, implementation, and evaluation of a DCTP, with emphasis on the context of the selected UoT. Thus, this stakeholder engagement will comprise (1) university’s academic executive management with representation from health sciences and envisioned collaborating faculties, such as engineering, accounting, and information systems; (2) key informants from universities with existing DCTPs, namely, the UKZN and Stellenbosch University; (3) external stakeholders from public sector and private sector health organizations with experiences of hosting DCTPs in their health establishments; (4) senior members of the student representative council for the FHS; and (5) heads of the academic departments identified for the pilot phase of the DCTP. Collectively, the stakeholders will provide valuable insights about the methods and approaches that will inform the piloting and establishment of a DCTP within the context of a UoT, particularly because such an initiative is novel within the context of the South African higher education sector. There is evidence suggesting that the implementation of a DCTP requires consideration of numerous factors including curriculum and pedagogy and various forms of resources including human, infrastructural, and financial resources, all of which have an impact on the sustainability of this transformative learning approach [1]. In the context of the DUT FHS, the existing practices and related sustainability elements are a product of collaborative engagement with various stakeholders from both the academic and nonacademic sector that ensure availability, scalability, and maintenance of the necessary faculty and university systems and processes that are required for teaching and learning. Thus, the combination of stakeholders is crucial for providing foundational insights about the practices and establishment of processes that will help in successfully piloting and establishing a DCTP beyond the pilot and initial implementation at the DUT FHS.

#### Step 3: Act and Observe

Following the stakeholder engagement workshops, a situational analysis of the university regarding the resources available, infrastructure, curriculum, teaching and learning, and pedagogical needs will be conducted, encompassing a multidisciplinary and multistakeholder involvement, whereas a narrative reporting process will be used to document findings, possible avenues of opportunity, recommendations and identification of possible communities, and organizations and health establishments that will be included as sites where decentralized clinical training will be provided. The stakeholders identified in step 2 will also be included in this phase, which will extend to the clinical placement facilities that will be identified as potential sites for the DCTP at the selected UoT.

#### Step 4: Reflection

This step will entail a consolidation of data obtained from steps 1 to 3 to inform the development of a conceptual framework to guide the development, pilot, evaluation, and establishment of the DCTP. In this step, the data generated for consolidation will be triangulated using a combination of theoretical models related to higher education pedagogy; teaching and learning practice frameworks; quality norms and standards related to teaching, learning, and assessment practices; and theories of program planning and implementation in health science education.

#### Step 5: Revised Plan

On the basis of the conceptual framework and policy documents emanating from step 4, strategic and operational systems related to the pilot phase of the DCTP will be formulated, with guidelines and operating procedures and standards to govern the teaching and learning practices, finance, procurement, and related operational activities essential for the pilot phase of the project.

### Phase 2: Empirical Phase

This phase will encompass a research and implementation science process, which will include the collection of primary data using multiple research designs and approaches to data collection. The multiple research designs will include qualitative case study design, descriptive qualitative design, phenomenography, narrative inquiry, and appreciative inquiry. The combination of individual interviews and focus group discussions will be used to collect data from participants through self-developed semistructured interview guides and open-ended questions. The design and implementation of interventions, systems, and processes for the piloting and implementation of the DCTP within the DUT FHS will be an integral part of this phase. Through ongoing consultation with relevant participants and stakeholders, the design, development, and establishment of the necessary infrastructure, human resources, pedagogical systems, financial systems, and related sustainability systems will be facilitated. Technological innovation for teaching and learning through biomedical engineering systems, telemedicine, and eHealth will also be a part of this phase, and the outcomes of this phase will be evaluated through the exploration of students and stakeholders’ experiences of using such technological applications in the context of teaching and learning within the DCTP at the DUT.

### Phase 3: Evaluation and Refining

#### Overview

This phase will encompass an implementation science research approach underpinned by action research and other research designs to facilitate the testing and evaluation of the DCTP within the selected pilot academic programs—namely, Clinical Technology, Radiography, and Emergency Medical Care—at the selected UoT in KwaZulu-Natal, South Africa. This phase will also entail primary data collection from stakeholders in relation to the outcomes of piloting the DCTP at the selected UoT and the assessment of established systems and processes to support the pilot phase of the DCTP as part of the testing and evaluation phase of the project. A combination of evaluation frameworks will be triangulated to guide the evaluation process using empirical research and implementation science approaches. These frameworks include models related to continuous quality improvement consistent with participatory action research and other impact evaluation frameworks.

#### Evaluation Framework

**Figure 1:**
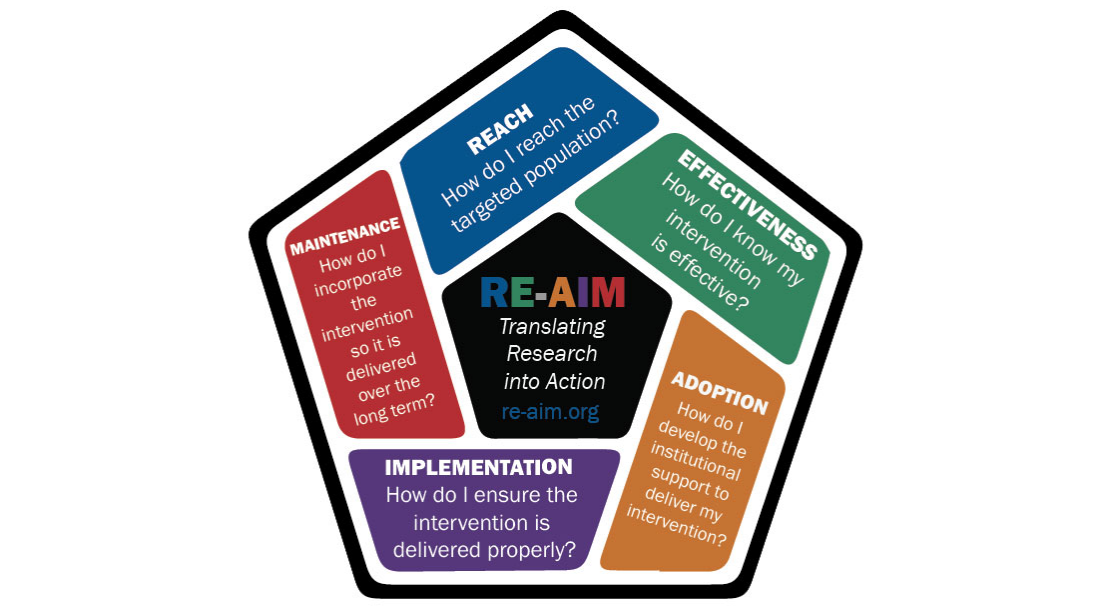
Elements of the Reach, Efficacy, Adoption, Implementation, and Maintenance (RE-AIM) framework.

The Reach, Efficacy, Adoption, Implementation, and Maintenance (RE-AIM) framework will be used to guide the evaluation of the pilot phase of the DCTP and its establishment at the selected UoT for primary data collection (Figure 1). The RE-AIM framework has been used in various contexts, ranging from clinical interventions to community programs, corporate settings, and multiple project phases from planning to implementation and summative evaluations [25]. The RE-AIM planning and evaluation framework is a tool for implementation and can be applied to assist in estimating the public impact of programs and interventions. Thus, it provides a structure to systematically evaluate program impact. It entails an explicit focus on issues relating to the design, dissemination, and implementation process of programs that can facilitate or impede success in achieving broad and equitable population-based impact. The framework also allows the use of both quantitative and qualitative methods to understand why and how the results were obtained for different RE-AIM dimensions. Thus, it will allow for the triangulation of different data sources and methods, which will both enhance the validity of the evaluation and allow for a better description of the generative mechanisms of the program. Multiple stakeholders will be included in data collection, drawing from qualitative and quantitative methods, using primary and secondary data. Accordingly, the evaluation will be conducted using nonsequential mixed methods, with equal importance attributed to the respective quantitative and qualitative components.

#### Application of the RE-AIM Evaluation Framework

The RE-AIM evaluation framework was selected to guide the evaluation process as it allows for comprehensive data collection using participatory mixed methods approaches to provide information about project outcomes. The envisaged multipronged approaches to facilitate piloting, implementation, and long-term sustainability of the DCTP at the DUT FHS will be placed within this framework. The elements of the RE-AIM framework, namely, (1) reach (2) effectiveness, (3) adoption, (4) implementation, and (5) maintenance, will be used as informed by their original theoretical definitions (Figure 1) to guide the formulation of questions that will be a part of the quantitative and qualitative data collection tools that will be used to generate routine data about the outcomes of DCTP based on the overarching objectives of this research project. The data generated from the evaluation process will inform interventions to ensure continuous quality improvement of the project to maintain the sustainability of the project. The precise questions that will be formulated as part of this process are yet to be developed; however, they relate to the outcomes of the project in terms of original intent; consistency; and standards of implementation, effectiveness, and long-term sustainability as aligned to the research objectives and elements of the framework.

### Phase 4: Establishment and Continuous Monitoring

This phase will encompass the implementation of the DCTP throughout all departments in the FHS, as informed by the collective results of phases 1 to 3 of the study. Primary data will be collected empirically from all identified stakeholders who would have been participants since phase 1 of the study. Quantitative and qualitative approaches will be used to collect data during this process regarding the outcomes of implementing DCTP as informed by various lived experiences, perceptions, and understanding of the phenomenon. Observational data will also be collected by conducting continuous assessments of the implementation processes in relation to specified standards, as guided by the conceptual and theoretical underpinnings informing the study. The quantitative and qualitative data will be triangulated by other sources of routine data as informed by the university and faculty standards and guidelines. This will subsequently inform practices related to continuous quality improvement and monitoring and evaluation. In addition, routine data about academic success indicators such as pass rates, graduation rates, and throughput rates will be collected. Consultative engagements will be facilitated with various industry personnel to determine the skills and graduate attributes of students who are trained through the DUT FHS DCTP project. This will be performed as part of the pilot, initial implementation phase and continuous monitoring during the eventual scale-up of the project.

### Data Analysis

#### Qualitative Data

Data analysis involves a systematic application of a process or processes for managing and organizing qualitative data, which brings order, structure, and meaning to the mass of data collected. Qualitative data analysis is an active process, whereby the researcher must carefully and deliberately scrutinize data that they have collected, often reading data repeatedly until meaning or deeper understanding of the data is obtained [26]. Analysis of qualitative data involves categorizing data into segments, with symbols or abbreviations used to classify words or phrases. This is known as coding [27,28]. In qualitative research studies, data analysis is performed concurrently with data collection and is conducted throughout the study [27]. Qualitative analysis techniques use words rather than numbers as a basis, and this strategy is contextual in nature. Analytical reasoning skills are required when conducting content analysis. In this study, data will be analyzed using the Tesch method of data analysis for qualitative research [28]. The steps in the Tesch method of data analysis include the following:

- Reading through all the transcripts carefully to obtain a sense of their content
- Picking 1 transcript randomly; going through it and asking oneself, “what is it about?”; and thinking about the underlying meaning of the interview
- Noting down thoughts in the margin
- Repeating this process with all the transcripts and making a list of all topics, and clustering similar topics together into columns, which consist of themes and subthemes
- Taking this list of topics and going back to the transcripts; abbreviating the topics as codes next to the appropriate segments of the text; and observing the organization of data to check whether new codes emerge

#### Quantitative Data

The data collected will be evaluated and analyzed using descriptive and inferential statistical methods in consultation with a statistician, using SPSS (version 26.0; IBM Corp). Descriptive statistical analysis for categorical data and continuous data will be presented as frequencies, percentages, and ranges. The descriptive statistics will be summarized in graphs and charts.

All electronic data will be stored in password-protected files, and hard-copy data will be stored in a locked cupboard that only the researcher has access to. Once the mandatory 5-year period after the study has passed, all electronic files will be deleted and all hard-copy data will be shredded.

#### Reliability and Validity

A pilot study will be conducted to test the feasibility of the survey questionnaire that will be developed as part of the quantitative data collection tools for the study. A statistician will be consulted to calculate the estimated study sample size for the quantitative data to be collected during the study, and logistic regression analysis will be used to ensure the reliability of the quantitative data.

#### Trustworthiness

Trustworthiness of the study will be operationalized through several methods to ensure rigor and robustness of the qualitative data that will be collected. Consistent with a social constructivist paradigm, the researcher will strive for the triangulation of data to ensure that multiple perspectives are captured. The researcher will accomplish data source triangulation by collecting data from various categories of stakeholders using multiple qualitative designs and data collection processes. Data method triangulation will be achieved by using different methods to collect data, namely, focus groups, semistructured interviews, and document review. Additional strategies will include peer debriefing and true member checking. To operationalize peer debriefing, the researcher will review the codes and themes with the study’s coinvestigators, which will allow for reflexivity and ensure that all the data are included. True member checking will entail summarizing the key points at the end of the focus groups and interviews and asking participants to verify the accuracy of the data. These strategies will ensure the credibility of the data. Dependability of the data will be achieved using an audit trail. The researcher will keep field notes from the participant interviews.

### Ethical Considerations

The basic principles of the Declaration of Helsinki will be adhered to throughout this research process. These include basic principles of respect, the right to self-determination, and the right to make informed decisions. All participants will sign an informed consent form after reading about the procedure of the study, indicating their willingness to participate in the study. The consent form highlights the benefits and risks of the study and requests permission to use audio recording in cases where interviews will be used to collect data. The right to withdraw at any stage of the research process will also be emphasized to participants. Beneficence will be ensured by supporting the well-being of participants during the study process. The study has received provisional ethical approval from the Durban University of Technology's Institutional Research Ethics Committee. Gatekeeper permission will be sought to access participants from the selected UoT and external universities identified for data collection. Moreover, gatekeeper permission will be obtained from the KwaZulu-Natal Department of Health, respective health facilities, and private sector health establishments that will be identified for data collection. Informed consent from individual participants will also be obtained before data collection.

## Results

The study has currently commenced with the situational analysis, consisting of engagement with external stakeholders implementing DCTPs. In this regard, engagement meetings have been held with the UKZN and Stellenbosch University. The situational analysis is to proceed with other stakeholders and will encompass formal data collection processes once full ethical approval has been obtained from the DUT. The data to be generated from completion of the situational analysis are anticipated to be published in 2024. The project will also be piloted and implemented in collaboration with the UKZN, a traditional university that has successfully implemented a DCTP and will provide valuable insights in terms of lessons and approaches that can be adopted by the DUT FHS. It is also envisaged that undergraduate students within the DUT FHS from the 4 selected departments will be placed in remote clinical facilities where the UKZN also places its students to facilitate interprofessional education. Moreover, students from the various departments at the DUT FHS will be placed collectively in locations where they do not share facilities with students from universities that are already implementing DCTP.

## Discussion

### Anticipated Findings

The DUT FHS functions with operational guidance underpinned by the institution’s ENVISION 2030 strategy and is guided by the evolving landscape of higher education, which is governed by local and international guidelines and best practices. The envisaged implementation of the DCTP at the UoT is in response to the existing gaps in implementation of DCTP at higher education institutions in South Africa. These gaps relate to the type of undergraduate health science students who are currently predominately trained through DCTPs, type of higher education institutions, and clinical placement facilities that are used for the practice of DCTPs. Studies suggest that training through DCTPs has largely been through traditional universities that offer mainstream biomedical health science disciplines in the field of medicine, nursing, optometry, occupational therapy, and physiotherapy. Health science disciplines such as medical orthotics and prosthetics, emergency medical care and rescue, radiography, dentistry, and dietetics and complementary health science disciplines remain neglected.

Literature also reveals that clinical placement facilities have largely been from the public health sector, with the use of community-based centers, primary health care facilities, and district hospitals as decentralized sites for learning. This is thus a gap in practice as the South African health system comprises public and private health care. In the context of the current implementation models of DCTPs, the use of remote private health facilities has not been reported in practice. The aforementioned gaps in existing practices of DCTPs and the nature of the UoT, in terms of teaching, learning, and assessment practices that differ from a traditional university, prompted this study. Moreover, the growing demand for clinical service placement, due to the increasing admissions of undergraduate health science students within the faculty, also necessitates the addition of new clinical placement facilities owing to a growing demand for such services in urban areas. Through the DUT FHS DCTP project, the growing demand for clinical service placement will be addressed through partnership with the ministry of health and private sector to increase the number of accredited health establishments that will allow for clinical service placement for students as part of their work-integrated learning and teaching practice of DCTPs. In this regard, an increasing number of distant health facilities will be sought through collaboration and cooperation with relevant authorities of these health establishments.

The reported benefits of DCTPs relate to the training outcomes of graduates regarding the mastery and skills required for the job as a health professional. In this regard, it is hoped that graduates within the UoT’s FHS will be better prepared for the current demands of the health system by virtue of the benefits of DCTPs in relation to learning experiences. This subsequently aligns with the university’s strategic objectives of producing creative, innovative, and adaptive graduates who will be able to make changes that lead to positive outcomes in the country and region. The DUT FHS remains anchored to its ENVISION 2030 strategy, which is founded upon four strategic perspectives, namely, (1) stewardship, (2) systems and processes, (3) sustainability, and (4) society, with the overarching vision of creativity, innovation, entrepreneurship, and production of adaptive graduates as outlined in the university’s statement of intent [29]. As DCTPs will facilitate authentic learning experiences through exposure to the real-world life experiences in varying clinical placement facilities, it will allow for the development of innovative and adaptive graduates as outlined in the university and faculty’s strategic objectives.

The changing landscape of service delivery caused by policy changes related to the globalization of health and the adoption of the principles of universal health coverage also require health science graduates who are responsive to local needs within a global view of health. As the DCTP creates learning opportunities that expose students to multiple realities of the health system, the implementation of the DCTP will contribute to producing more fit-for-purpose graduates who are able to better respond to the diverse health needs of communities based on varying geographic and sociocultural contexts. This will be realized through clinical placement of students in diverse health care facilities that are located away from the university, thereby allowing for exposure to real-life experiences that form a part of the learning process. Therefore, this contributes to the knowledge and skill set required for health graduates to provide optimal health care services based on the needs of communities. Thus, the DCTP contributes to improved competencies and skill set of graduates in this manner. The exposure to different health establishments that are distant from the university, encompassing private and public health facilities, will reveal the differences in health systems’ functioning and establishment and will enhance learning by virtue of the inherent diversity, thus contributing to adaptive graduates who are more informed about the varying complexities of the South African national health system that is underpinned by the principles of universal health coverage.

Another major gap identified in the current practice of DCTPs is the lack of integration between private sector and public sector health facilities during the training of students. The new NHI policy will require graduates who will be able to adapt easily to the demands of both private and public sectors, as an integral part of the policy involves partnership with the private sector to facilitate optimum standards of health service delivery for all. Thus, the design, piloting, evaluation, and establishment of the DCTP at the DUT will include the element of collaboration between the private and public sectors. The outcomes of this collaboration will include improved skill sets for health science graduates, and they will be exposed to the dynamics of the private and public health sectors as clinical placement will be conducted in health establishments in the private and public health sectors. This may contribute to more well-rounded health professionals in terms of the operational functioning of the health systems in the 2 sectors. Moreover, students will be exposed to more diverse clinical health situations, which will form part of their learning process.

Partnership with the KwaZulu-Natal Department of Health, the private sector, and the UKZN are foundational elements for the successful implementation of this project. The collaboration between these entities is novel, due to the distinct differences in organizational structure and strategic mandate. The differences in primary purpose, policy orientation, and operations among the DUT, UKZN, private health sector, and public health sector is one of the many key factors that are anticipated to pose as challenges to the successful scale-up and establishment of the DCTP at the DUT FHS. These organizational differences may hinder the teaching practices that may be adopted to facilitate learning for undergraduate health science students and may contribute to unforeseen interruptions that may have implications for implementation of the project. Ongoing engagement and communication with all stakeholders at various levels within the 4 establishments is planned as one of the key strategies for mitigating the possible challenges that may arise. The development of an institutional DCTP steering committee with representatives from all 4 establishments is also planned as an approach for mitigating the anticipated challenges. The collaboration among the DUT, UKZN, private sector, and public sector will also be formalized through a documented, joint memorandum of agreement, which will be signed by all the relevant parties for legal and ethical purposes and for mitigating the reported and anticipated challenges. Despite the distinct differences and anticipated challenges of such a partnership, this collaboration will generate new insights about how institutions with historically diverse philosophies can cooperate with each other for the purpose of transforming health science education, fostering interprofessional and collaborative practice, and improving health service delivery.

Currently, no formal collaboration exists among the UoT, UKZN, Department of Health, and the private sector; however, due to the availability of existing data about the implementation of a DCTP at UKZN, the possibility of a successful rollout with a UoT is a notion that is not farfetched. Formal guidelines to operationalize the processes and guide the execution of the project are currently unavailable and are thus necessary in light of the unique dimensions within which the project will be implemented. Thus, the following recommendations provide foundational principles for operationalizing the DCTP at the DUT and will guide the project’s execution:

- *Ongoing multilevel stakeholder engagement and collaboration*: This will allow the generation of comprehensive primary data to inform findings related to the research process, which will ultimately be holistically integrated into data that will inform the operationalization of the DCTP during the pilot, implementation, and eventual scale-up.
- *Resource allocation*: For the piloting, implementation, and scale-up of the project to be achieved, financial, human, and physical resources will have to be provided. The allocation of financial resources is most critical as it will ensure that the necessary infrastructure, personnel, and processes are instituted to enable the success of the project.
- *Relevant policy and system review, development, and realignment*: As one of the foundational elements of this project is collaboration with different stakeholders and entities, the review of existing policies and guidelines governing the organization’s operational process will be necessary to ensure the seamless integration of the DUT with the identified partner organizations.
- *Curriculum review and realignment*: Existing practices related to teaching and learning within the undergraduate health science programs at the DUT FHS will have to be reviewed, and findings of the review process will inform the realignment of the curriculum to match the dynamics within the clinical placement facilities, thereby producing graduates who are adaptive and responsive to the health needs of the communities and society, as informed by experiences that are encountered in the decentralized clinical placement facilities.
- *Continuous professional development and mentorship*: Academic and other relevant stakeholders within the FHS and DUT should receive training, mentorship, and support regarding the recommended teaching, learning, and assessment practices to be adopted within the context of a DCTP. This should be aligned with the relevant institutional policy and appropriate local and international best practices. This may facilitate buy-in from the stakeholders who will be responsible for implementation, thereby ensuring the success of the project.
- *Availability of monitoring, evaluation, and continuous quality assurance processes*: This may be in the form of ≥1 tool such as guidelines, standard operating procedures, audit tools, monitoring and evaluation framework, and routine data collection instruments that monitor the progress of the project at pilot, implementation, and scale-up phases to ensure its success in terms of the overarching objectives. Facilitating ongoing scholarship related to DCTP implementation within a UoT and its operational process will be an integral part of the broad project and the monitoring and evaluation process to ensure continuous quality improvement as supported by empirical evidence.
- *Facilitating technological innovation*: A key aspect of implementation of the DCTP within the DUT FHS is technological innovation for teaching and learning, which will have to be embedded within the teaching practices. In this regard, IT systems, infrastructure, and applications will have to be developed, sourced, and continuously updated to ensure compliance with innovative teaching practices. Therefore, an integral part of the implementation process is the development of systems rooted in biomedical engineering, telemedicine, and eHealth technologies.

The adoption of recommended foundational principles for the operationalization and execution of the DUT FHS DCTP project has implications for pilot, scale-up, and sustainability of the project beyond implementation. Moreover, the long-term sustainability of the project will be ensured through the development of a clear road map for the DCTP that provides short-term, intermediate, and long-term guidance for the execution of the project. The project is integrated into the DUT FHS long-term strategic and operational plans as a flagship initiative. This ensures the availability of financial, human, and physical resources necessary for the sustainability of the project beyond its pilot and initial implementation phases. The FHS strategic operational plan with the embedded DCTP project will subsequently form a part of the institutional strategic plan, thus further ensuring long-term sustainability.

### Conclusions

It is envisioned that the DUT FHS DCTP project will facilitate collaboration among the UoTs, traditional universities, Ministry of Health, and private sector for clinical placement of undergraduate health science students in health establishments that are away from the university, thereby exposing them to real-life experiences related to health care. This will facilitate authentic learning experiences that will contribute to improved competencies of graduates in relation to the health needs of the society and the multiple realities of the South African health system.

## Abbreviations

DCTP: Decentralized Clinical Training Program
DUT: Durban University of Technology
FHS: Faculty of Health Sciences
NHI: National Health Insurance
RE-AIM: Reach, Efficacy, Adoption, Implementation, and Maintenance
UKZN: University of KwaZulu-Natal
UoT: university of technology

## Data Availability

Data sharing is not applicable to this paper as no data sets were generated or analyzed during this study.
